# Extreme Antibiotic Persistence via Heterogeneity-Generating Mutations Targeting Translation

**DOI:** 10.1128/mSystems.00847-19

**Published:** 2020-01-21

**Authors:** Anupama Khare, Saeed Tavazoie

**Affiliations:** a Department of Biological Sciences, Columbia University, New York, New York, USA; b Department of Systems Biology, Columbia University, New York, New York, USA; c Department of Biochemistry and Molecular Biophysics, Columbia University, New York, New York, USA; University of Massachusetts Medical School

**Keywords:** *Escherichia coli*, antibiotic persistence, gene expression heterogeneity, laboratory evolution, persisters, systems biology, translation

## Abstract

Antibiotic persistence, the noninherited tolerance of a subpopulation of bacteria to high levels of antibiotics, is a bet-hedging phenomenon with broad clinical implications. Indeed, the isolation of bacteria with substantially increased persistence rates from chronic infections suggests that evolution of hyperpersistence is a significant factor in clinical therapy resistance. However, the pathways that lead to hyperpersistence and the underlying cellular states have yet to be systematically studied. Here, we show that laboratory evolution can lead to increase in persistence rates by orders of magnitude for multiple independently evolved populations of Escherichia coli and that the driving mutations are highly enriched in translation-related genes. Furthermore, two distinct adaptive mutations converge on concordant transcriptional changes, including increased population heterogeneity in the expression of several genes. Cells with extreme expression of these genes showed dramatic differences in persistence rates, enabling isolation of subpopulations in which a substantial fraction of cells are persisters. Expression analysis reveals coherent regulation of specific pathways that may be critical to establishing the hyperpersistence state. Hyperpersister mutants can thus enable the systematic molecular characterization of this unique physiological state, a critical prerequisite for developing antipersistence strategies.

**IMPORTANCE** Bacterial persistence is a fascinating phenomenon in which a small subpopulation of bacteria becomes phenotypically tolerant to lethal antibiotic exposure. There is growing evidence that populations of bacteria in chronic clinical infections develop a hyperpersistent phenotype, enabling a substantially larger subpopulation to survive repeated antibiotic treatment. The mechanisms of persistence and modes of increasing persistence rates remain largely unknown. Here, we utilized experimental evolution to select for Escherichia coli mutants that have more than a thousandfold increase in persistence rates. We discovered that a variety of individual mutations to translation-related processes are causally involved. Furthermore, we found that these mutations lead to population heterogeneity in the expression of specific genes. We show that this can be used to isolate populations in which the majority of bacteria are persisters, thereby enabling systems-level characterization of this fascinating and clinically significant microbial phenomenon.

## Abstract (precis)

Bacterial persistence is a fascinating phenomenon in which a small subpopulation of bacteria becomes phenotypically tolerant to lethal antibiotic exposure. There is growing evidence that populations of bacteria in chronic clinical infections develop a hyperpersistent phenotype, enabling a substantially larger subpopulation to survive repeated antibiotic treatment. The mechanisms of persistence and modes of increasing persistence rates remain largely unknown. Here, we utilized experimental evolution to select for Escherichia coli mutants that have more than a thousandfold increase in persistence rates. We discovered that a variety of individual mutations to translation-related processes are causally involved. Furthermore, we found that these mutations lead to population heterogeneity in the expression of specific genes. We show that this can be used to isolate populations in which the majority of bacteria are persisters, thereby enabling systems-level characterization of this fascinating and clinically significant microbial phenomenon.

## INTRODUCTION

The phenomenon of antibiotic persistence, in which a small fraction of a bacterial population can survive exposure to high levels of antimicrobials, is thought to enable the rise of antibiotic resistance (1). Such persistence has been observed in almost 30 bacterial species, as well as in several fungi (2). Persister cells can survive in the presence of antibiotic concentrations severalfold higher than the MIC of the population (3). The level of persistence can vary by several orders of magnitude depending on the bacterial species, physiological state or growth phase of the population, and the antibiotic used. The frequency of persisters can range from 0.001% to almost the entire population being “persistent” in the case of stationary-phase Escherichia coli populations against β-lactams, as well as stationary-phase Staphylococcus aureus populations against most antibiotics (2, 4). While persistence was classically thought to be a phenomenon in which subpopulations of cells are tolerant to most antibiotics, recent studies have shown that persistence levels vary widely in the presence of diverse antibiotics, and changes in levels of persistence due to mutations or environmental fluctuations can be specific to one or a few antibiotics (2).

Over the past few decades, mutations in several loci that affect the persistence level of bacterial populations have been identified (5, 6), and several putative mechanisms underlying persistence have been described (7). Clinical strains with high persistence levels have also been isolated from chronic infections (8, 9), suggesting that repeated exposure to antibiotics could select for hyperpersistence. Laboratory evolution experiments have shown similar increases in the persistence levels of bacterial populations (10–12), but the diversity of adaptive pathways and the breadth of underlying transcriptional modulations that can lead to hyperpersistence have not been systematically studied. Here, we used laboratory experimental evolution of multiple independent E. coli populations to study the emergence of hyperpersistence, identify the driving mutations that lead to increased phenotypic heterogeneity, and discover molecular markers that enable isolation and characterization of the hyperpersisting subpopulations.

## RESULTS

### Experimental evolution of hyperpersistence is mediated by translation-related alleles.

A major class of molecules that has been implicated in persistence in multiple bacterial species is the toxin-antitoxins (TA) (13), although the role of some TA modules in E. coli persistence has recently been questioned (14). We were interested in identifying upstream global regulators and pathways that affected persistence rates and in elucidating adaptive trajectories that would access the mutational space of central cellular processes, and hence chose to study these in bacteria lacking many of the known TA modules (15). While E. coli contains more than 30 TA systems (16), the 10 type II TA systems had previously been implicated in playing a significant role in persistence, and hence we chose to use a previously constructed strain that lacked these TA modules.

**FIG 1:**
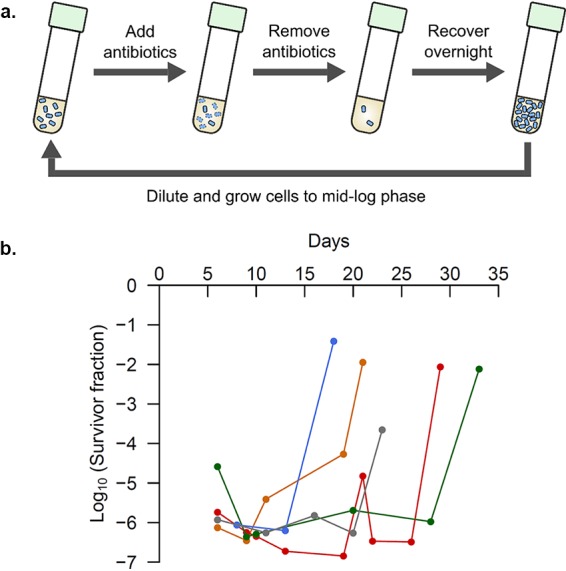
Experimental evolution of hyperpersistence. (a) Selection for increased levels of persistence was carried out as shown. Populations of E. coli cells were grown to the mid-log phase and exposed to lethal levels of two different classes of antibiotics to select for persisters. The antibiotics were then removed, and the persisters were allowed to recover overnight. Such selection cycles were repeated daily until the populations showed substantially increased levels of persistence. (b) The persistence levels of five populations that were independently evolved against ampicillin and ciprofloxacin are shown at various time points during the laboratory evolution of hyperpersistence.

We deleted the *hipBA* locus in the Δ*10TA* strain (15), which lacks the other 10 type II toxin-antitoxin modules present in E. coli. This strain, which we refer to here as the Δ*TA11* strain, was subjected to daily selections for persister cell survival (Fig. 1a). We exposed these cells to repeated cycles of two antibiotics with orthogonal modes of action (the combination of either ampicillin plus ciprofloxacin or ampicillin plus kanamycin) in order to avoid selecting for antibiotic resistance and to evolve general hyperpersistence independent of the antibiotic mechanism of action. Additionally, our selection was carried out in the exponential phase of growth to provide insights into persistence in actively growing bacterial populations.

Multiple independently evolved populations attained substantially higher levels of persistence after varying numbers of selection cycles (Fig. 1b), at which point the selection was stopped. We isolated three individual clones from each of the evolved populations that had significantly higher persistence rates (most showed a more than 1,000-fold increase), but similar resistance, compared to the parental strain. Most of the evolved isolates had the same MICs as the parental strain for both antibiotics used for the selection, whereas a few had a 2-fold higher MIC, which is considered to be within the acceptable deviation for MIC measurements (17)—only 5/33 strains for ampicillin, 2/24 for ciprofloxacin, and 1/9 for kanamycin had one or both replicate MIC measurements 2-fold higher than the MIC for the parental strain. Our experimental evolution was performed using two antibiotics in combination, to avoid selection of resistance, which is illustrated by the MICs for the evolved strains being similar to those for the parental strain. However, antibiotics are known to interact with each other, affecting both the rate of killing (18) and the evolution of resistance (19). We therefore tested for persistence in subsequent experiments with only one antibiotic, to avoid such confounding effects. The individual clones were subjected to whole-genome sequencing, which led to the identification of several mutations not present in the parental strain. We excluded the analysis of cryptic prophage genes since the original Δ*10TA* strain was shown to harbor several mutations in these genes (20), which may confound the interpretation of any additional evolved alleles. All of the remaining identified mutations are listed in Data Set S1.

10.1128/mSystems.00847-19.9 DATA SET S1 All mutations identified in the evolved isolates (except for prophage-associated mutations). Download Data Set S1, XLSX file, 0.02 MB. Copyright © 2020 Khare and Tavazoie. 2020 Khare and Tavazoie This content is distributed under the terms of the Creative Commons Attribution 4.0 International license.

To increase the likelihood of identifying the driving mutations that underlie the evolved hyperpersistence phenotype, we focused on mutant alleles common to all three isolates from each evolved population (Table S1). Several of these mutations were in genes involved in tRNA functionality during translation, and, given the potentially global effect of these alleles, we chose to focus on this class of mutations. These included mutations that led to single amino acid changes or small insertions/deletions in different tRNA synthetase enzymes (IleS, LeuS, MetG, and ProS), which catalyze the attachment of amino acids to their cognate tRNAs (21). We found three independent mutations resulting in small deletions in the C terminus of the MetG protein, which we had previously shown via transposon mutagenesis studies to cause increased persistence (5). Additionally, a previous lab evolution study had found that a single amino acid change in *metG* leads to an extended lag time, and hence increased persistence, since the bulk of the population remains dormant at the time of antibiotic treatment (10). We further studied one of the *metG* mutations, a 12-bp deletion (positions 1726 to 1737 in the coding region), here referred to as metG*. We also found two independent mutations in the *pth* gene (referred to here as pth* [G101D] and pth1* [A170P]), both leading to single amino acid substitutions. The essential gene *pth* codes for the enzyme peptidyl tRNA hydrolase, which functions to cleave peptidyl-tRNAs that are prematurely released from the ribosome, thus recycling tRNAs in the cell (22). One of the mutations, pth* (G101D), had been previously identified as a temperature-sensitive allele in a study characterizing the *pth* locus (23).

10.1128/mSystems.00847-19.6 TABLE S1 Mutations seen in all 3 sequenced isolates for each population. The mutations in bold were related to tRNA function in translation and were studied further. Download Table S1, PDF file, 0.2 MB. Copyright © 2020 Khare and Tavazoie. 2020 Khare and Tavazoie This content is distributed under the terms of the Creative Commons Attribution 4.0 International license.

**FIG 2:**
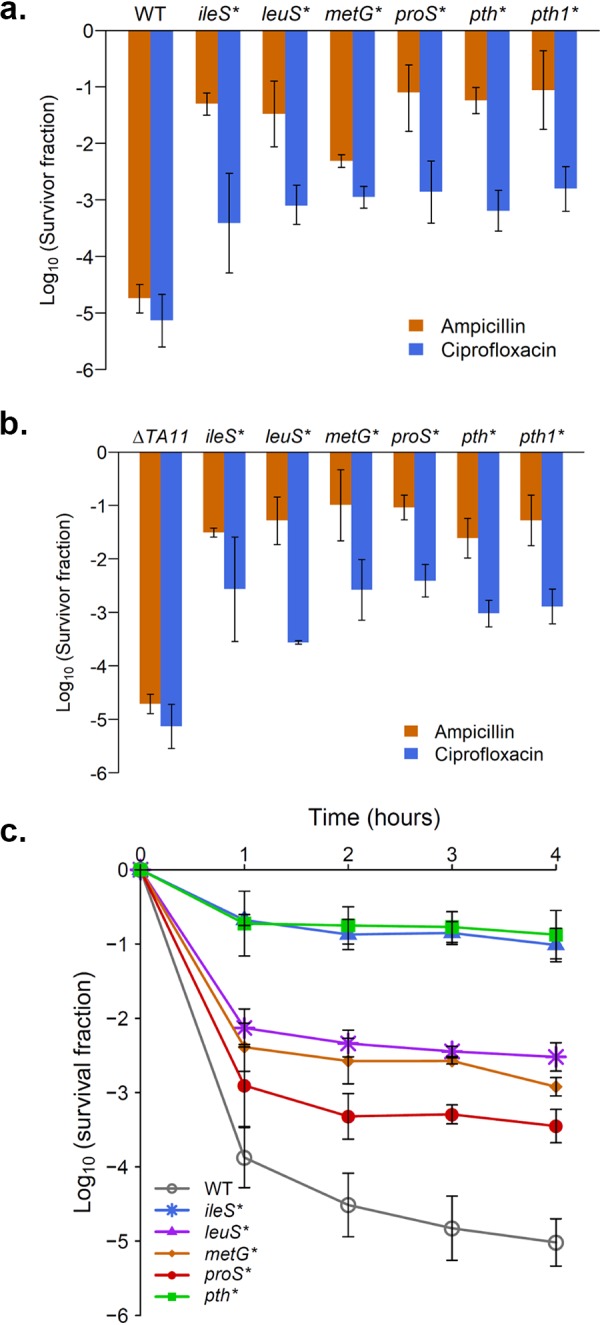
Evolved tRNA-related alleles confer hyperpersistence. The persistence levels of strains carrying the evolved alleles in the WT MG1655 and the Δ*TA11* backgrounds were measured against ampicillin and ciprofloxacin. All of the alleles shown led to increased levels of persistence compared to the parental background. Shown are the means of at least three replicate experiments, with the error bars depicting the standard deviation. The survival of the strains 4 h after antibiotic exposure was measured in both the (a) MG1655 and (b) Δ*TA11* backgrounds. All of the mutant values are significantly different from those for the corresponding parental strain, as tested by a one-way analysis of variance (ANOVA) followed by a Tukey’s test (*P* < 0.001). (c) The mutant strains showed the characteristic biphasic killing that is associated with persistence, as measured in the MG1655 background against ampicillin. All of the 4-h mutant values are significantly different from those for the parental strain, as tested by a one-way ANOVA followed by a Tukey’s test (*P* < 0.0001).

We transferred these mutations singly to both the wild-type (WT) MG1655 and Δ*TA11* backgrounds, and we observed a substantial increase in their persistence rates against both ampicillin and ciprofloxacin (Fig. 2a and b). Despite the strains being lysogenized by the phage Φ80 (14), and the absence of the 11 type II TA systems in the Δ*TA11* background, we saw only minor differences in persistence levels between the two backgrounds. Strains with the mutant alleles had MICs similar to those for the parental background (Table S2), and they also showed the biphasic killing characteristic of persistence (Fig. 2c), indicating that these mutant alleles conferred increased persistence and not resistance or tolerance. Similarly to previously identified hyperpersister mutants (10), most of our strains carrying the individual evolved alleles had longer lag phases but similar maximal growth rates in the exponential phase compared to the WT (Table S2). However, they retained their hyperpersistent phenotype even when the bulk population was in the exponential phase of growth (Fig. S2). We also found a mutation in the *selU* gene, which is involved in the modification of selenium-containing tRNAs (24), but it did not confer increased persistence in isolation (Fig. S1), and hence was not studied further. Since the Δ*10TA* strain was found to be infected with the common lab phage contaminant Φ80 (14), and to have multiple secondary mutations (20), we carried out all of our subsequent experiments in an MG1655 (WT) background. In order to test for Φ80 contamination in our strains, we used a cross-streak assay with Φ80 lysate, and we observed that none of the strains in the MG1655 background were lysogenized by Φ80, whereas the ones in the Δ*10TA* background were (data not shown).

10.1128/mSystems.00847-19.1 FIG S1 The selU* allele does not lead to increased persistence. Strains carrying the evolved selU* allele did not confer high persistence against either ampicillin or ciprofloxacin in the (a) wild-type (WT) MG1655 or (b) Δ*TA11* backgrounds. Shown are the means of at least four replicate experiments, with the error bars depicting the standard deviation. In all cases, persistence of the selU* mutants was not significantly different from that of the parental strain, as tested by the Wilcoxon rank sum test (*P* > 0.9). Download FIG S1, PDF file, 0.1 MB. Copyright © 2020 Khare and Tavazoie. 2020 Khare and Tavazoie This content is distributed under the terms of the Creative Commons Attribution 4.0 International license.

10.1128/mSystems.00847-19.2 FIG S2 The allele replacement strains have increased persistence at different phases of growth. WT and allele replacement strains were tested for persistence against (a) ampicillin and (b) ciprofloxacin at various initial cell densities (between 10^7^ and 10^9^ CFU/ml). The gray dots show the WT persistence, and the colored dots show the persistence of the respective allele replacement mutants (the WT data are shown in each panel for easy visual comparison). The evolved alleles led to high persistence at all initial cell densities. All of the mutants are significantly different from the WT (*P* < 0.05), as determined by pairwise Wilcoxon rank sum tests followed by the Benjamini-Hochberg correction for multiple testing. Download FIG S2, PDF file, 0.1 MB. Copyright © 2020 Khare and Tavazoie. 2020 Khare and Tavazoie This content is distributed under the terms of the Creative Commons Attribution 4.0 International license.

10.1128/mSystems.00847-19.7 TABLE S2 MICs, doubling time, and lag time of the strains carrying the adaptive alleles. Download Table S2, PDF file, 0.1 MB. Copyright © 2020 Khare and Tavazoie. 2020 Khare and Tavazoie This content is distributed under the terms of the Creative Commons Attribution 4.0 International license.

### Evolved hyperpersistent alleles increase population variability for persistence markers.

**FIG 3:**
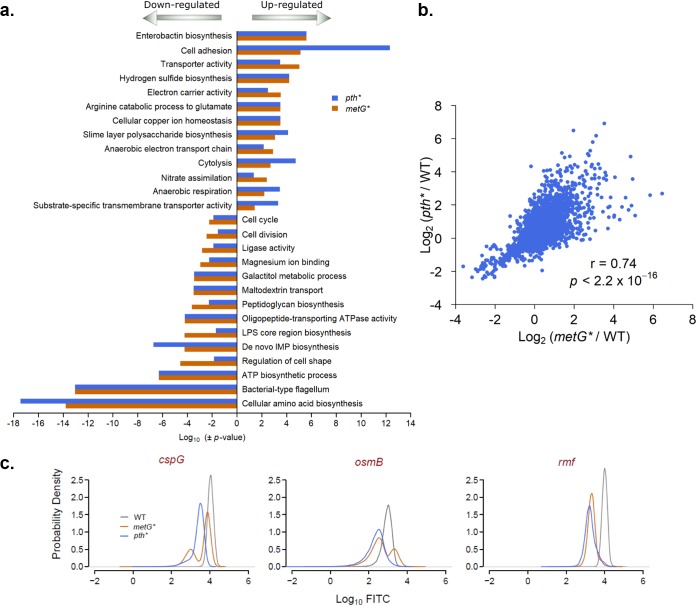
Hyperpersistent mutants show similar reprogramming of gene expression. The mRNA expression of all genes in WT, metG*, and pth* bacterial populations was determined in the exponential phase. (a) The changes in gene expression for all genes in the exponential phase were determined for the metG* and pth* mutants compared to the WT. For both mutants, the genes were ordered by their fold change relative to the WT and divided into five equal sized bins. The functional gene ontology (GO) categories that were significantly enriched or depleted in these bins (*P* < 0.05; hypergeometric) were identified using iPAGE (output shown in Fig. S5). Shown here are the *P* values for the GO categories that are significantly enriched in either the most upregulated (negative log_10_ of the *P* values) or the most downregulated bin (log_10_ of the *P* values). The positive values show the upregulated categories, and the negative values the downregulated ones. (b) The fold change in gene expression in the metG* and pth* mutants compared to the parental WT strain was highly correlated. (c) We measured the fluorescence of WT, metG*, and pth* strains carrying a green fluorescent protein (GFP) reporter under the expression of promoters of some of the genes that were the most overexpressed in the mutants. Shown here are the promoters that showed a broader or bimodal distribution in at least one of the hyperpersistent mutants.

The adaptive alleles lead to substantially higher levels of persistence, suggesting that at least a significant subpopulation of cells in these multiple strains likely exists in a distinct physiological state characterized by the differential expression of one or more cellular pathways. We therefore determined bulk global gene expression changes in both the metG* and pth* mutants compared to the WT to delineate two independent gene expression states of hyperpersistence (Data Set S2). We focused on these mutants since multiple adaptive populations had converged on mutations in these genes, suggesting that they may be key modulators for the evolution of hyperpersistence. To identify the pathways that are divergent in each of the mutants compared to the WT, we used iPAGE, a mutual-information based tool for pathway analysis that we used previously to analyze gene functional enrichment across transcriptional profiling data (25). Remarkably, we observed a high overlap in the pathways that were significantly up- and downregulated in the two mutants (Fig. 3a and Fig. S5). In both mutants, we saw a significant enrichment of genes involved in the cell cycle as well as the biosynthesis of ATP, amino acids, nucleotides, lipopolysaccharide (LPS), peptidoglycan, and the flagellum among the most highly repressed genes. The coordinated downregulation of several core biosynthetic pathways indicates that either the entire hyperpersistent population or a significant subpopulation might exist in a catabolic, stress-responsive physiological cellular state in both hyperpersistent mutants. The concordance in pathway deregulation was maintained at the gene level, as we observed a highly significant correlation (*P* value < 2.2 × 10^−16^) between the global change in gene expression in the two mutants compared to the WT (Fig. 3b). This suggested that common global changes in gene expression may underlie the emergence of increased persistence by both adaptive alleles.

**FIG 4:**
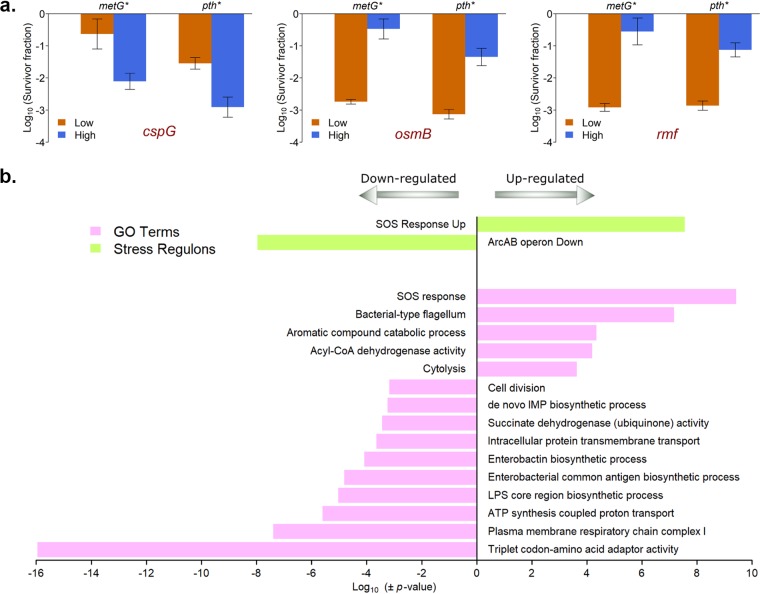
Persister-enriched cells in the metG* mutant have a well-defined transcriptional state. (a) metG* and pth* mutants expressing GFP under the control of different promoters were sorted into two populations having 5% of the cells with the highest and 5% of the cells with the lowest fluorescence. The persistence level of these sorted populations against ampicillin was then determined. Cells expressing the lowest fluorescence for *cspG* and the highest fluorescence for *rmf* and *osmB* were associated with high persistence in both the metG* and the pth* mutants. Shown are the means of three replicate experiments, with the error bars depicting the standard deviation. The high and low populations are significantly different from each other in all cases, as tested by paired *t* tests (*P* < 0.05). (b) Subpopulations of the metG* mutant expressing p-*osmB*-GFP with high fluorescence (>33% survival) and low fluorescence (<0.6% survival) were sorted, and global gene expression measurements were carried out using transcriptome sequencing (RNA-seq). Genes were ordered by the fold change in gene expression in the high-persister population compared to the low-persister population, and then divided into 5 equally sized bins. Cellular pathways and stress regulons that were significantly enriched or depleted in these bins (*P* < 0.05) were identified using iPAGE. Shown here are the *P* values for the categories most highly enriched (*P* < 0.001) in the most upregulated (−log_10_[*P* value]) and downregulated (log_10_[*P* value]) bins. The positive values represent the upregulated categories, and the negative values the downregulated categories. The entire iPAGE output is shown in Fig. S5. CoA, coenzyme A.

10.1128/mSystems.00847-19.10 DATA SET S2 Normalized expression values obtained from transcriptional profiling of the WT and the metG* and pth* mutants (associated with Fig. 3a and b), as well as from the transcriptional profiling of the sorted high- and low-GFP fluorescence populations from metG* p-*osmB* (associated with Fig. 4b). Download Data Set S2, XLSX file, 0.5 MB. Copyright © 2020 Khare and Tavazoie. 2020 Khare and Tavazoie This content is distributed under the terms of the Creative Commons Attribution 4.0 International license.

Differential regulation of genes in the bulk population may reflect pathways whose activity affects the frequency of persister cells. Additionally, some of the changes in gene expression may also reflect population heterogeneity due to the presence of a relatively high fraction of persisters. Some of these common differentially expressed genes may thus represent specific markers for a shared persistent cellular state in the hyperpersistent mutants. To test this, we examined the population distributions of gene expression for some of the genes that were most differentially regulated and had at least moderate levels of expression in both metG* and pth*. We utilized plasmids from a library of fluorescent transcriptional reporters that had been previously constructed in E. coli (26). We measured the fluorescence level of individual cells in bacterial populations of the WT, metG*, and pth* strains carrying these plasmids (Fig. S3). Interestingly, several genes (*rmf*, *osmB*, and *cspG*) showed either a bimodal or broader population fluorescence distribution in at least one of the mutants (Fig. 3c), suggesting that one or both mutations were leading to increased variability of gene expression, at least for these specific markers. The population distributions did not necessarily match the bulk gene expression data, likely due to the fact that the fluorescence reporters are not subject to the same posttranscriptional regulation as the native transcripts.

10.1128/mSystems.00847-19.3 FIG S3 Some of the most highly over- or underexpressed genes in hyperpersistent strains show population distributions with increased variability compared to the WT. The fluorescence of WT and hyperpersistent mutant populations without a green fluorescent protein (GFP) construct (a) or carrying a GFP reporter under the expression of differentially expressed promoters was measured. We tested promoters of some of the most overexpressed genes (b) and the most underexpressed genes (c). Several promoters (*rmf*, *cspG*, *osmB*, and *hdeA)* had increased variability in at least one of the hyperpersistent mutants. Download FIG S3, PDF file, 0.1 MB. Copyright © 2020 Khare and Tavazoie. 2020 Khare and Tavazoie This content is distributed under the terms of the Creative Commons Attribution 4.0 International license.

Increased heterogeneity in the expression of specific genes in the hyperpersistent mutant populations suggested that these genes might serve as markers for persistence in these strains. To test this hypothesis, we sorted bacterial populations of the WT, metG*, and pth* strains bearing fluorescent reporter plasmids (26) for *rmf*, *osmB*, and *cspG* into subpopulations containing 5% of cells having the highest and 5% having the lowest fluorescence, and then tested the persistence levels of these sorted populations (Fig. 4a). For the parental WT strain, persistence levels were too low to be quantified from the sorted populations. However, in both the metG*, and pth* mutants, the highest expression of *rmf* and *osmB* and the lowest expression of *cspG* were associated with substantially higher levels of persistence. In the metG* mutant, we obtained subpopulations in which a high proportion of the population consisted of persisters (survival varied from 11 to 81% in the individual replicates). Interestingly, even though *cspG* was significantly overexpressed in bulk RNA measurements in both hyperpersistent mutants, increased persistence was associated with lower levels of *cspG* expression. However, this matched the population distribution in the metG* mutant (Fig. 3c) that showed a small subpopulation of cells with lower fluorescence, which was likely enriched for persisters. This reiterates the fact that population heterogeneity underlies the persistence phenotype, and studying this at the level of individual cells is crucial to gain insight into the molecular state of persister cells (27). As a control, we also sorted similarly for two genes whose expression was not significantly different between the WT and the mutants and observed no difference in persistence levels (Fig. S4).

10.1128/mSystems.00847-19.4 FIG S4 Expression of genes that are not differentially expressed in hyperpersistent mutants is not associated with difference in persistence levels. Populations of hyperpersister mutants expressing GFP under the control of either the (a) *lepA* or (b) *speE* promoters were sorted into two different populations, consisting of the 5% of the cells with the highest and 5% of the cells with the lowest fluorescence. The persistence rates of these sorted populations were then determined. Shown are the means of three replicate experiments, with the error bars depicting the standard deviation. The persistence levels of the sorted populations were not significantly different, as tested by a paired *t* test (*P* > 0.1). Download FIG S4, PDF file, 0.1 MB. Copyright © 2020 Khare and Tavazoie. 2020 Khare and Tavazoie This content is distributed under the terms of the Creative Commons Attribution 4.0 International license.

### The transcriptional state of persister-enriched subpopulations indicates cellular stress.

The molecular state of persister cells has been difficult to characterize due to the small fraction of persisters present in a bacterial population and the associated challenges in isolating this subpopulation. To determine genes and pathways that are differentially expressed between the persisting and nonpersisting subpopulations, we measured the transcriptional profile of the 5% of metG* cells expressing the highest levels of the *osmB* reporter (subpopulations with 43% and 50% persister cells in the two biological replicates that were used for transcriptome sequencing [RNA-seq]) and compared it to the 5% of cells with the lowest levels of *osmB* reporter expression (<0.6% persister cells in both replicates) (Data Set S2). Interestingly, gene ontology (GO) term enrichment analysis showed that this subpopulation has a well-defined cellular state characterized by upregulation of the SOS response, phage shock, and flagellar genes and the downregulation of genes involved in core processes such as the electron transport chain, ATP production, translation (tRNAs), LPS biosynthesis, purine biosynthesis, cell division, and protein transport (Fig. 4b and Fig. S5). Downregulation of several of these pathways was also seen in the bulk mRNA expression in metG* and pth* mutants (Fig. 3a), further corroborating the fact that these are associated with the persister state in these mutants. Additionally, similar enrichment analysis on stress regulons supported the GO term results by revealing the upregulation of the SOS response and the downregulation of genes repressed by the ArcAB two-component system (which are commonly involved in aerobic respiration) in the mutants (Fig. 4b and Fig. S5).

10.1128/mSystems.00847-19.5 FIG S5 Outputs from iPAGE runs on transcriptional profiling data. Shown are iPAGE runs on the fold change in gene expression in the metG* and pth* mutants compared to the WT (gene ontology [GO] terms), as well as the fold change in gene expression in the high-GFP subpopulation from metG* p-*osmB* compared to the low-GFP subpopulation (GO terms and stress regulons). Download FIG S5, PDF file, 0.1 MB. Copyright © 2020 Khare and Tavazoie. 2020 Khare and Tavazoie This content is distributed under the terms of the Creative Commons Attribution 4.0 International license.

## DISCUSSION

Our work demonstrates that bacterial populations can rapidly attain high levels of persistence via multiple adaptive trajectories that converge on genes involved in protein translation, a key cellular process in all organisms. Phenomena similar to persistence have been seen in a variety of bacterial species, as well as in fungi, protozoa, and even drug-tolerant tumor subpopulations (28), and it is possible that adaptive trajectories targeting translation may also play a role in those systems. A major priority for understanding persistence is the characterization of the unique physiological state of persister cells in terms of the expression and activity of genes, proteins, and metabolites. However, the low frequency of persistence (<10^−4^) has made this a challenging endeavor. In our study, mutations in two genes involved in different aspects of translation (*metG* and *pth*) both lead to hyperpersistent populations with common transcriptional states. Several of the most differentially expressed genes serve as markers for the persistent state, and extreme expression of these is associated with subpopulations in which up to 80% of the cells are persisters. Identification of the transcriptional regulators of these marker genes may thus reveal central modulators of the persistent cellular state. Furthermore, these marker genes enabled purification of highly enriched populations of persisters, providing unique insights into their gene expression states. Persister cells in these mutants are characterized by increased expression of genes involved in stress response pathways such as the SOS response and the phage shock response, as well as by decreased expression of most core cellular metabolic pathways, a combination of which may underlie their tolerance to antibiotic exposure.

Perhaps the most fascinating question arising from our work is the mechanism(s) by which diverse genetic perturbations to tRNA functionality and translation give rise to heterogeneity in gene expression states that clearly underlie persistence. Despite persistence being a phenomenon of phenotypic heterogeneity, hyperpersistent alleles that increase variability in the expression of persistence-associated markers have not been described before. It is possible that the tRNA synthetase mutations lead to decreased translational fidelity, potentially due to alterations in the proofreading activity of the tRNA synthetases (29). The *pth* mutations likely affect the availability of free tRNAs in the cell and could lead to reduced translational fidelity due to mischarging of the tRNAs similar to what happens during amino acid starvation (30). Such error-prone translation may lead to population heterogeneity and result in a substantial fraction of cells that experience cellular stress and downregulate core anabolic pathways. The physiological state of these cells could recapitulate several mechanisms of persistence that have been previously described, such as reduced levels of ATP (20, 31), activation of the stringent response (32) or the SOS response (33), accumulation of active toxins (34) or protein aggregates (35), and reduced growth rate (36).

These are important areas to explore in future work. The combination of laboratory experimental evolution and systems biological studies, as presented here, promises to advance our basic understanding of persistence and establish a rational foundation for development of antipersistence strategies in the clinic.

## MATERIALS AND METHODS

### Strains and growth conditions.

All strains and plasmids used in this study are described in Table S3.

10.1128/mSystems.00847-19.8 TABLE S3 Bacterial strains, plasmids, and primers used in this study. Download Table S3, PDF file, 0.1 MB. Copyright © 2020 Khare and Tavazoie. 2020 Khare and Tavazoie This content is distributed under the terms of the Creative Commons Attribution 4.0 International license.

For all experiments in liquid medium, bacteria were grown at 37°C and shaken at 300 rpm in supplemented M9 media containing 6 g/liter Na_2_HPO_4_, 3 g/liter KH_2_PO_4_, 0.5 g/liter NaCl, 1 g/liter NH_4_Cl, 2 mM MgSO_4_, 0.5 mM CaCl_2_, 0.4% glucose, 2 μM ferric citrate, 1× Supplement EZ (Teknova), 1× ACGU solution (Teknova), and the micronutrient supplement described by Neidhardt et al. (37). For routine strain construction, strains were grown in LB (10 g/liter Bacto tryptone, 5 g/liter yeast extract, and 10 g/liter NaCl) supplemented with an antibiotic (50 μg/ml kanamycin, 50 μg/ml chloramphenicol, or 100 μg/ml ampicillin), if necessary.

### Laboratory evolution of hyperpersistence.

A previous study (14) pointed out several concerns about protocols commonly used to measure persistence due to the resulting variability in determined persistence levels. Several of our experimental parameters adhere to the recommended methods—we used high concentrations of antibiotics (200 μg/ml ampicillin, 5 μg/ml ciprofloxacin, or 200 μg/ml kanamycin) for our experimental evolution and our persistence assays, since intermediate antibiotic levels could have confounding effects due to prophage induction. Furthermore, there were concerns raised about significant differences in persister phenotypes in different media. All of our experiments were carried out in a rich defined M9-based medium to avoid any variability associated with complex medium or with any potential nutritional dependencies of mutants that might arise during the evolution. Additionally, it had been shown that in a defined medium such as M9, the number of persister cells for both ampicillin and ciprofloxacin remained stable for several hours until the cells enter the late exponential phase. In our experiments, antibiotic exposure occurs prior to the late exponential phase, suggesting that we are measuring the stable levels of persisters seen in the exponential phase.

Δ*TA11* E. coli cells were diluted 1:100 from overnight culture (grown for 17 to 18 hours) into 2 ml of fresh medium, and then grown for 2 hours. Antibiotics (either 200 μg/ml ampicillin and 5 μg/ml ciprofloxacin or 200 μg/ml ampicillin and 200 μg/ml kanamycin) were added to the cells, and the cells were treated for 4 h of shaking at 300 rpm at 37°C. Cells were then washed twice with 1× phosphate-buffered saline (PBS), resuspended in 2 ml of medium, and grown overnight for 17 to 18 h. The long overnight growth meant that the cultures likely reached similar cell densities prior to the next inoculum. This process was repeated for 10 to 44 days, and persistence levels were tested at intervals of a few days. Of all the measurements of persistence levels, 46 out of 47 populations had an initial cell density (at the time of antibiotic treatment) of 1 × 10^7^ to 4.6 × 10^8^ CFU/ml, suggesting that almost all of the populations were in exponential phase. The one remaining population had an initial cell density of 4.5 × 10^6^ CFU/ml, which likely reflects either biological or technical variability. The evolution was stopped when persistence levels significantly higher than the parental population were seen. Aliquots of the evolving populations were frozen after the overnight growth. We tested the persistence levels and MICs of individual clones from the evolved populations and proceeded with whole-genome sequencing of clones with significantly higher persistence levels, but similar MICs, compared to the parental strain.

### Whole-genome sequencing of evolved strains.

Indexed paired-end libraries were prepared for sequencing the genomic DNA of evolved strains as previously described (38). The samples were pooled, then sequenced on an Illumina NextSeq 500 sequencer for 75 cycles. The data were analyzed as described (38). Briefly, the sequencing reads were preprocessed using Cutadapt (39) and Trimmomatic (40), and the mutations present in the evolved strains compared to the parental background were identified using breseq v0.26 (41).

### Generation of allele replacement strains and the Δ*TA11* strain.

The *hipBA* locus was deleted in the Δ*10TA* strain (15) using the one-step inactivation method (42). The kanamycin resistance cassette from plasmid pKD4 (42) was amplified with primers that had 40 bp of homology to regions upstream and downstream of the *hipBA* locus. The PCR product was electroporated into Δ*10TA* cells carrying the pKD46 plasmid (42), in which expression of λ Red recombinase had been induced with 1.5 mM arabinose. Transformants with the correct insertion were identified by PCR, and the kanamycin cassette was excised using the pCP20 plasmid (42) as previously described (38). Evolved alleles were transferred using the pKOV plasmid (43, 44) as previously described (38). The primers used for strain construction are listed in Table S3.

### Persistence assays.

Overnight cultures of bacteria were diluted 1:100 in fresh medium and grown for 2 h with shaking at 300 rpm and 37°C. An aliquot was diluted in 1× PBS and spotted on LB plates to determine the initial cell density. The appropriate antibiotic was added to this culture (200 μg/ml ampicillin or 5 μg/ml ciprofloxacin), and the cultures were treated at 37°C at 300 rpm for 4 h. Cells were washed twice and diluted with 1× PBS, then spotted on LB plates to determine the final cell density. For the kill curves shown in Fig. 2c, cells were similarly spotted after 1, 2, 3, and 4 h. The experiments shown in Fig. S2 were performed similarly, except that cells were grown in fresh medium for 1, 2, or 3 hours prior to addition of the antibiotic to get a range of initial cell densities. Shown in the figure are the data where the initial cell densities were between 10^7^ to 10^9^ CFU/ml.

For the persistence assays on the sorted populations, 10^4^ cells from each subpopulation were sorted into 0.5 ml of supplemented M9 media, and an aliquot was removed for measuring the initial cell density. Ampicillin (200 μg/ml) was then added to the cells, and the cultures were treated at 37°C at 300 rpm for 3 h. Cells were washed twice with 1× PBS, and dilutions were plated on LB plates to determine the final cell density.

### Measurement of growth rates.

Overnight cultures of bacteria were diluted 1:200 in 150 μl of fresh medium and grown in a BioTek Synergy Mx plate reader shaking continuously for 24 hours at 37°C. The absorbance at 600 nm (OD_600_) was measured every 10 minutes. Doubling times and lag phase lengths were calculated using the grofit package in R (45).

### Measurement of MICs.

Overnight cultures of bacteria were diluted 1:200 in 150 μl of supplemented M9 medium containing a dilution series of the antibiotics (1 to 512 μg/ml ampicillin, or 0.0039 to 2 μg/ml ciprofloxacin), and grown for 24 hours at 37°C. The MIC was determined as the minimum antibiotic concentration where no growth of the bacterial culture was seen.

### Transcriptional profiling.

Overnight cultures of bacteria were diluted 1:100 in fresh medium and grown at 300 rpm at 37°C, until they reached an OD_600_ of 0.3. At this time, 2 ml of the culture was mixed with 4 ml of RNAprotect bacteria reagent (Qiagen), incubated at room temperature for 5 min, and centrifuged at 5,500 × *g* for 10 minutes. After discarding the supernatant, the pellets were stored at −80°C. Total RNA was made from the pellets using the Total RNA Purification Plus kit (Norgen) according to the manufacturer’s recommended protocol for Gram-negative bacteria.

rRNA was removed from these samples using the Ribo-Zero rRNA removal kit (Illumina). We then prepared indexed libraries from the resulting RNA using the NEBNext Ultra directional RNA library prep kit for Illumina. The samples were pooled and sequenced on a NextSeq 500 sequencer for 75 cycles.

For the transcriptional profiling from the sorted populations, 2× RNAprotect bacteria reagent was added to the sorted cells, samples were incubated at room temperature for 5 min, centrifuged at 16,000 × *g* for 10 minutes, and the pellets were stored at −80°C. The pellets were resuspended in 500 μl TRIzol reagent (Invitrogen) and 100 μl chloroform and mixed thoroughly. The aqueous phase was removed after centrifuging for 5 min at 12,000 × *g* at 4°C in Phasemaker tubes (Invitrogen). Ethanol (3× the volume) was added to the aqueous phase, and RNA was purified from that using a Zymo RNA Clean and Concentrator-5 kit.

Illumina adapter sequences were removed from the reads using Cutadapt (39), and the ends of the reads were trimmed to eliminate poor quality bases using Trimmomatic (40). We aligned the reads to the genome using Bowtie 2 (46), mapped them to individual genes using BEDTools (47), and determined reads per kilobase per million (RPKM) values for each. We then calculated the mean for each strain from two biological replicates, and sum normalized the counts across the three strains. We set a threshold of the fifth percentile of WT gene expression (0.498), and all counts lower than the threshold were set to that level. These normalized expression values are listed in Data Set S2. We used these values to calculate the fold change in gene expression in the mutants compared to the WT.

We determined the enrichment and depletion of pathways among the most up- and downregulated genes in the mutants using iPAGE (25). We ran iPAGE in continuous mode with 5 bins and included all of the redundant GO categories or transcription factor regulons in order to identify pathways deregulated in the mutants compared to the parental WT strain. In Fig. 3a, we have shown the common upregulated and downregulated categories, with the redundant ones removed. The full iPAGE output figures are in Fig. S5.

For the sorted populations, we preprocessed the samples and mapped to individual genes as before. We then removed all reads that mapped to the rRNA genes, and then calculated the RPKM values and the mean for each subpopulation from two biological replicates. We sum normalized the counts across the two populations and set a threshold of the fifth percentile of low green fluorescent protein (GFP) gene expression (1.01), and all counts lower than the threshold were set to that level. These normalized expression values are listed in Data Set S2. We ran iPAGE for the sorted populations, using 5 bins to test for enrichment of GO terms and stress regulons, to obtain Fig. S5. We have shown the most enriched categories (*P* < 0.001) in Fig. 4b.

### Flow cytometry and sorting on marker genes.

For the flow cytometry experiments, we first picked the top 20 genes showing the highest (or lowest) fold enrichment in each of the mutants (metG* and pth*) that had at least moderate levels of expression in WT as well as in both mutants, in order to enable measurement and comparison of population fluorescence distributions across the three strains. We set a minimum threshold of sum-normalized RPKM values greater than 10 in all three strains. We then selected several of these genes depending on the availability of promoter constructs and by restricting our choice to only one gene per operon. We then measured the population distributions of expression for several genes from these lists. The flow cytometry and sorting were carried out at the National Cancer Institute (NCI) Center for Cancer Research (CCR) Flow Cytometry Core. Bacterial cells were grown to the exponential phase (OD_600_ = 0.15 to 0.3), diluted in PBS, and then analyzed by flow cytometry using a Sony SA3800 instrument, using gates that include the majority of the single-cell population. While the use of PBS for diluting E. coli cells for flow cytometry has been recently called into question due to an increase in cell death and increased variability in fluorescence distributions (48), the increased variation and bimodality we see is restricted to specific promoters in only the mutant strains, suggesting that it is associated with the mutant alleles and is not an artifact of using PBS. The GFP fluorescence was measured for 30,000 events, using a fluorescein isothiocyanate (FITC) laser/filter. The average fluorescence intensity of WT, *metG*, and *pth* strains without a GFP construct was calculated as a measure of bacterial autofluorescence, and this value was then subtracted from each measurement for the promoter reporters in the appropriate strain to obtain background-corrected values. For the data shown in Fig. 3C and Fig. S3, the probability density of the background-corrected fluorescence intensity distributions for each population was plotted using R. We used the “density” function in R (with the default parameters), which uses kernel density estimation for smoothing, to determine the probability density function.

For the sorting on the expression of the promoters of the marker genes, bacterial cells were similarly grown to the exponential phase and then sorted on a BD FACS Aria Fusion cell sorter. The forward and side scatter of the bacterial populations were measured, and the populations were gated to avoid clumps and debris. The populations were then gated into bins of cells having the highest 5% and lowest 5% of GFP fluorescence. In total, 100,000 cells from each bin were sorted into 0.5 ml of supplemented M9 medium. For the transcriptional profiling of the sorted populations, at least 1 million cells were similarly sorted into each bin.

## References

[1] Levin-Reisman I, Ronin I, Gefen O, Braniss I, Shoresh N, Balaban NQ 2017 Antibiotic tolerance facilitates the evolution of resistance. Science 355: 826–830. doi:10.1126/science.aaj2191. 28183996

[2] Van den Bergh B, Fauvart M, Michiels J 2017 Formation, physiology, ecology, evolution and clinical importance of bacterial persisters. FEMS Microbiol Rev 41:219–251. doi:10.1093/femsre/fux001. 28333307

[3] Balaban NQ, Helaine S, Lewis K, Ackermann M, Aldridge B, Andersson DI, Brynildsen MP, Bumann D, Camilli A, Collins JJ 2019 Definitions and guidelines for research on antibiotic persistence. Nat Rev Microbiol 17:441–448. doi:10.1038/s41579-019-0196-3. 30980069 PMC7136161

[4] Conlon BP 2014 *Staphylococcus aureus* chronic and relapsing infections: evidence of a role for persister cells. Bioessays 36:991–996. doi:10.1002/bies.201400080. 25100240

[5] Girgis HS, Harris K, Tavazoie S 2012 Large mutational target size for rapid emergence of bacterial persistence. Proc Natl Acad Sci U S A 109:12740–12745. doi:10.1073/pnas.1205124109. 22802628 PMC3411964

[6] Harms A, Maisonneuve E, Gerdes K 2016 Mechanisms of bacterial persistence during stress and antibiotic exposure. Science 354:aaf4268. doi:10.1126/science.aaf4268. 27980159

[7] Wilmaerts D, Windels EM, Verstraeten N, Michiels J 2019 General mechanisms leading to persister formation and awakening. Trends Genet 35:401–411. doi:10.1016/j.tig.2019.03.007. 31036343

[8] Mulcahy LR, Burns JL, Lory S, Lewis K 2010 Emergence of *Pseudomonas aeruginosa* strains producing high levels of persister cells in patients with cystic fibrosis. J Bacteriol 192:6191–6199. doi:10.1128/JB.01651-09. 20935098 PMC2981199

[9] LaFleur MD, Qi Q, Lewis K 2010 Patients with long-term oral carriage harbor high-persister mutants of *Candida albicans*. Antimicrob Agents Chemother 54:39–44. doi:10.1128/AAC.00860-09. 19841146 PMC2798516

[10] Fridman O, Goldberg A, Ronin I, Shoresh N, Balaban NQ 2014 Optimization of lag time underlies antibiotic tolerance in evolved bacterial populations. Nature 513:418–421. doi:10.1038/nature13469. 25043002

[11] Mechler L, Herbig A, Paprotka K, Fraunholz M, Nieselt K, Bertram R 2015 A novel point mutation promotes growth phase-dependent daptomycin tolerance in *Staphylococcus aureus*. Antimicrob Agents Chemother 59:5366–5376. doi:10.1128/AAC.00643-15. 26100694 PMC4538524

[12] Van den Bergh B, Michiels JE, Wenseleers T, Windels EM, Boer PV, Kestemont D, De Meester L, Verstrepen KJ, Verstraeten N, Fauvart M 2016 Frequency of antibiotic application drives rapid evolutionary adaptation of *Escherichia coli* persistence. Nat Microbiol 1:16020. doi:10.1038/nmicrobiol.2016.20. 27572640

[13] Page R, Peti W 2016 Toxin-antitoxin systems in bacterial growth arrest and persistence. Nat Chem Biol 12:208. doi:10.1038/nchembio.2044. 26991085

[14] Harms A, Fino C, Sørensen MA, Semsey S, Gerdes K 2017 Prophages and growth dynamics confound experimental results with antibiotic-tolerant persister cells. mBio 8:e01964-17. doi:10.1128/mBio.01964-17. 29233898 PMC5727415

[15] Maisonneuve E, Shakespeare LJ, Jørgensen MG, Gerdes K 2011 Bacterial persistence by RNA endonucleases. Proc Natl Acad Sci U S A 108:13206–13211. doi:10.1073/pnas.1100186108. 21788497 PMC3156201

[16] Yamaguchi Y, Park J-H, Inouye M 2011 Toxin-antitoxin systems in bacteria and archaea. Annu Rev Genet 45:61–79. doi:10.1146/annurev-genet-110410-132412. 22060041

[17] Mouton JW, Muller AE, Canton R, Giske CG, Kahlmeter G, Turnidge J 2017 MIC-based dose adjustment: facts and fables. J Antimicrob Chemother 73:564–568. doi:10.1093/jac/dkx427. 29216348

[18] Yeh P, Tschumi AI, Kishony R 2006 Functional classification of drugs by properties of their pairwise interactions. Nat Genet 38:489. doi:10.1038/ng1755. 16550172

[19] Hegreness M, Shoresh N, Damian D, Hartl D, Kishony R 2008 Accelerated evolution of resistance in multidrug environments. Proc Natl Acad Sci U S A 105:13977–13981. doi:10.1073/pnas.0805965105. 18779569 PMC2544564

[20] Shan Y, Gandt AB, Rowe SE, Deisinger JP, Conlon BP, Lewis K 2017 ATP-dependent persister formation in *Escherichia coli*. mBio 8:e02267-16. doi:10.1128/mBio.02267-16. 28174313 PMC5296605

[21] Pang YLJ, Poruri K, Martinis SA 2014 tRNA synthetase: tRNA aminoacylation and beyond. Wiley Interdiscip Rev RNA 5:461–480. doi:10.1002/wrna.1224. 24706556 PMC4062602

[22] Das G, Varshney U 2006 Peptidyl-tRNA hydrolase and its critical role in protein biosynthesis. Microbiology 152:2191–2195. doi:10.1099/mic.0.29024-0. 16849786

[23] García‐Villegas MR, De La Vega F, Galindo J, Segura M, Buckingham R, Guarneros G 1991 Peptidyl‐tRNA hydrolase is involved in lambda inhibition of host protein synthesis. EMBO J 10:3549–3555. doi:10.1002/j.1460-2075.1991.tb04919.x. 1833189 PMC453084

[24] Wolfe MD, Ahmed F, Lacourciere GM, Lauhon CT, Stadtman TC, Larson TJ 2004 Functional diversity of the rhodanese homology domain the *Escherichia coli ybbB* gene encodes a selenophosphate-dependent tRNA 2-selenouridine synthase. J Biol Chem 279:1801–1809. doi:10.1074/jbc.M310442200. 14594807

[25] Goodarzi H, Elemento O, Tavazoie S 2009 Revealing global regulatory perturbations across human cancers. Mol Cell 36:900–911. doi:10.1016/j.molcel.2009.11.016. 20005852 PMC2900319

[26] Zaslaver A, Bren A, Ronen M, Itzkovitz S, Kikoin I, Shavit S, Liebermeister W, Surette MG, Alon U 2006 A comprehensive library of fluorescent transcriptional reporters for *Escherichia coli*. Nat Methods 3:623. doi:10.1038/nmeth895. 16862137

[27] Balaban NQ, Merrin J, Chait R, Kowalik L, Leibler S 2004 Bacterial persistence as a phenotypic switch. Science 305:1622–1625. doi:10.1126/science.1099390. 15308767

[28] Cohen NR, Lobritz MA, Collins JJ 2013 Microbial persistence and the road to drug resistance. Cell Host Microbe 13:632–642. doi:10.1016/j.chom.2013.05.009. 23768488 PMC3695397

[29] Mohler K, Ibba M 2017 Translational fidelity and mistranslation in the cellular response to stress. Nat Microbiol 2:17117. doi:10.1038/nmicrobiol.2017.117. 28836574 PMC5697424

[30] Parker J 1989 Errors and alternatives in reading the universal genetic code. Microbiol Rev 53:273. 2677635 10.1128/mr.53.3.273-298.1989 PMC372737

[31] Conlon BP, Rowe SE, Gandt AB, Nuxoll AS, Donegan NP, Zalis EA, Clair G, Adkins JN, Cheung AL, Lewis K 2016 Persister formation in *Staphylococcus aureus* is associated with ATP depletion. Nat Microbiol 1:16051. doi:10.1038/nmicrobiol.2016.51. PMC4932909 27572649

[32] Amato SM, Fazen CH, Henry TC, Mok WW, Orman MA, Sandvik EL, Volzing KG, Brynildsen MP 2014 The role of metabolism in bacterial persistence. Front Microbiol 5:70. doi:10.3389/fmicb.2014.00070. 24624123 PMC3939429

[33] Dörr T, Lewis K, Vulić M 2009 SOS response induces persistence to fluoroquinolones in *Escherichia coli*. PLoS Genet 5:e1000760. doi:10.1371/journal.pgen.1000760. 20011100 PMC2780357

[34] Schumacher MA, Balani P, Min J, Chinnam NB, Hansen S, Vulić M, Lewis K, Brennan RG 2015 HipBA–promoter structures reveal the basis of heritable multidrug tolerance. Nature 524:59. doi:10.1038/nature14662. 26222023 PMC7502270

[35] Pu Y, Li Y, Jin X, Tian T, Ma Q, Zhao Z, Lin S-y, Chen Z, Li B, Yao G 2019 ATP-dependent dynamic protein aggregation regulates bacterial dormancy depth critical for antibiotic tolerance. Molecular Cell 73:143–156.e4. doi:10.1016/j.molcel.2018.10.022. 30472191

[36] Pontes MH, Groisman EA 2019 Slow growth determines nonheritable antibiotic resistance in *Salmonella enterica*. Sci Signal 12:eaax3938. doi:10.1126/scisignal.aax3938. 31363068 PMC7206539

[37] Neidhardt FC, Bloch PL, Smith DF 1974 Culture medium for enterobacteria. J Bacteriol 119:736–747. doi:10.1128/JB.119.3.736-747.1974. 4604283 PMC245675

[38] Khare A, Tavazoie S 2015 Multifactorial competition and resistance in a two-species bacterial system. PLoS Genetics 11:e1005715. doi:10.1371/journal.pgen.1005715. 26647077 PMC4672897

[39] Martin M 2011 Cutadapt removes adapter sequences from high-throughput sequencing reads. EMBnet J 17:10–12. doi:10.14806/ej.17.1.200.

[40] Bolger AM, Lohse M, Usadel B 2014 Trimmomatic: a flexible trimmer for Illumina sequence data. Bioinformatics 30:2114–2120. doi:10.1093/bioinformatics/btu170. 24695404 PMC4103590

[41] Deatherage DE, Barrick JE 2014 Identification of mutations in laboratory-evolved microbes from next-generation sequencing data using breseq, p 165–188. In Sun L, Shou W (ed), Engineering and analyzing multicellular systems. Springer, New York, NY. 10.1007/978-1-4939-0554-6_12 PMC4239701 24838886

[42] Datsenko KA, Wanner BL 2000 One-step inactivation of chromosomal genes in *Escherichia coli* K-12 using PCR products. Proc Natl Acad Sci U S A 97:6640–6645. doi:10.1073/pnas.120163297. 10829079 PMC18686

[43] Bulyk ML, McGuire AM, Masuda N, Church GM 2004 A motif co-occurrence approach for genome-wide prediction of transcription-factor-binding sites in *Escherichia coli*. Genome Res 14:201–208. doi:10.1101/gr.1448004. 14762058 PMC327095

[44] Link AJ, Phillips D, Church GM 1997 Methods for generating precise deletions and insertions in the genome of wild-type *Escherichia coli*: application to open reading frame characterization. J Bacteriol 179:6228–6237. doi:10.1128/jb.179.20.6228-6237.1997. 9335267 PMC179534

[45] Kahm M, Hasenbrink G, Lichtenberg-Fraté H, Ludwig J, Kschischo M 2010 grofit: fitting biological growth curves with R. J Stat Softw 33:1–21. doi:10.18637/jss.v033.i07.20808728

[46] Langmead B, Salzberg SL 2012 Fast gapped-read alignment with Bowtie 2. Nat Methods 9:357. doi:10.1038/nmeth.1923. 22388286 PMC3322381

[47] Quinlan AR, Hall IM 2010 BEDTools: a flexible suite of utilities for comparing genomic features. Bioinformatics 26:841–842. doi:10.1093/bioinformatics/btq033. 20110278 PMC2832824

[48] Tomasek K, Bergmiller T, Guet CC 2018 Lack of cations in flow cytometry buffers affect fluorescence signals by reducing membrane stability and viability of *Escherichia coli* strains. J Biotechnol 268:40–52. doi:10.1016/j.jbiotec.2018.01.008. 29355812

