# Supplementary material for: Extreme Antibiotic Persistence via Heterogeneity-Generating Mutations Targeting Translation
Source: mSystems. 2020 Jan 21;5(1):e00847-19. doi: 10.1128/mSystems.00847-19 (PMC6977076; doi:10.1128/mSystems.00847-19)
Supplement: TABLE S1 [file mSystems.00847-19-st001.pdf]

Table S1

Selection	Population	Position	Mutation	Annotation	Gene
Ampicillin + Ciprofloxacin	TA11_AC1	**673,433**	**A→G**	**F192L (TTT→CTT)**	***leuS***
		**673,937**	**A→C**	**F24V (TTT→GTT)**	***leuS***
		2,545,720	Δ41 bp	coding (1026-1066/1425 nt)	*murP*
	TA11_AC2	**530,085**	**T→G**	**E122D (GAA→GAC)**	***selU***
		**2,194,047**	**Δ12 bp**	**coding (1726-1737/2034 nt)**	***metG***
	TA11_AC3	3,678,797	Δ3 bp	coding (1165-1167/1497 nt)	*yhjJ*
	TA11_AC4	303,522	T→C	intergenic (-116/+197)	*ykgJ/ecpE*
		303,524	A→G	intergenic (-118/+195)	*ykgJ/ecpE*
		303,531	T→C	intergenic (-125/+188)	*ykgJ/ecpE*
		433,892	G→T	V8F (GTT→TTT)	*ribE*
		**1,257,229**	**C→G**	**A170P (GCG→CCG)**	***pth***
		1,949,227	A→T	I124I (ATA→ATT)	*yecD*
		3,990,826	Δ6 bp	coding (1651-1656/2547 nt)	*cyaA*
	TA11_AC5	1,949,227	A→T	I124I (ATA→ATT)	*yecD*
		**2,194,122**	**Δ18 bp**	**coding (1801-1818/2034 nt)**	***metG***
	TA11_AC6	**22,835**	**(TGCTGGGCG)1→2**	**coding (445/2817 nt)**	***ileS***
		303,522	T→C	intergenic (-116/+197)	*ykgJ/ecpE*
		303,524	A→G	intergenic (-118/+195)	*ykgJ/ecpE*
		303,526	C→G	intergenic (-120/+193)	*ykgJ/ecpE*
		303,531	T→C	intergenic (-125/+188)	*ykgJ/ecpE*
		1,949,227	A→T	I124I (ATA→ATT)	*yecD*
	TA11_AC7	303,522	T→C	intergenic (-116/+197)	*ykgJ/ecpE*
		1,656,600	Δ1 bp	coding (508/2427 nt)	*ynfE*
		1,949,227	A→T	I124I (ATA→ATT)	*yecD*
		**2,194,226**	**Δ6 bp**	**coding (1905-1910/2034 nt)**	***metG***
	TA11_AC8	**218,652**	**A→T**	**W42R (TGG→AGG)**	***proS***
		303,522	T→C	intergenic (-116/+197)	*ykgJ/ecpE*
		303,524	A→G	intergenic (-118/+195)	*ykgJ/ecpE*
		303,526	C→G	intergenic (-120/+193)	*ykgJ/ecpE*
		1,949,227	A→T	I124I (ATA→ATT)	*yecD*
		2,031,969	C→A	intergenic (-461/-106)	*yedR/yedS*
		2,421,051	G→A	R171C (CGC→TGC)	*yfcI*
Ampicillin + Kanamycin	TA11_AK1	2,310,124	A→C	G216G (GGT→GGG)	*ompC*
		4,293,047	C→A	R182S (CGT→AGT)	*gltP*
	TA11_AK2	**1,257,435**	**C→T**	**G101D (GGC→GAC)**	***pth***
	TA11_AK3	458,014	IS186 (+) +6 bp :: Δ1	intergenic (+90/-93)	*clpX/lon*
		**1,257,435**	**C→T**	**G101D (GGC→GAC)**	***pth***
