# Supplementary figures and images for: Extreme Antibiotic Persistence via Heterogeneity-Generating Mutations Targeting Translation
Source: mSystems. 2020 Jan 21;5(1):e00847-19. doi: 10.1128/mSystems.00847-19 (PMC6977076; doi:10.1128/mSystems.00847-19)

Figure S1

a.

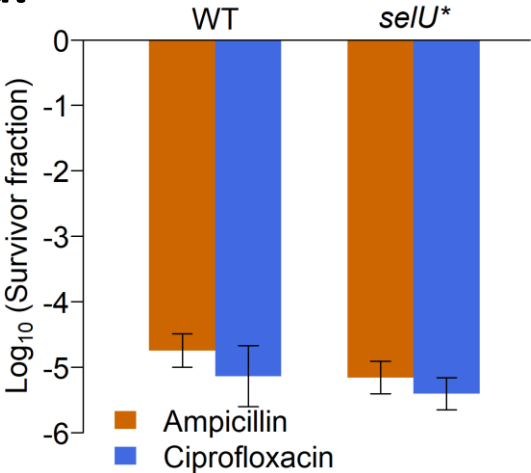

b.

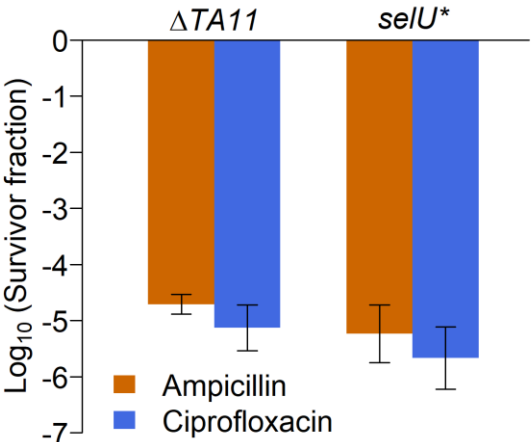

Supplement: FIG S1 [file mSystems.00847-19-sf001.pdf]

Figure S2

a.

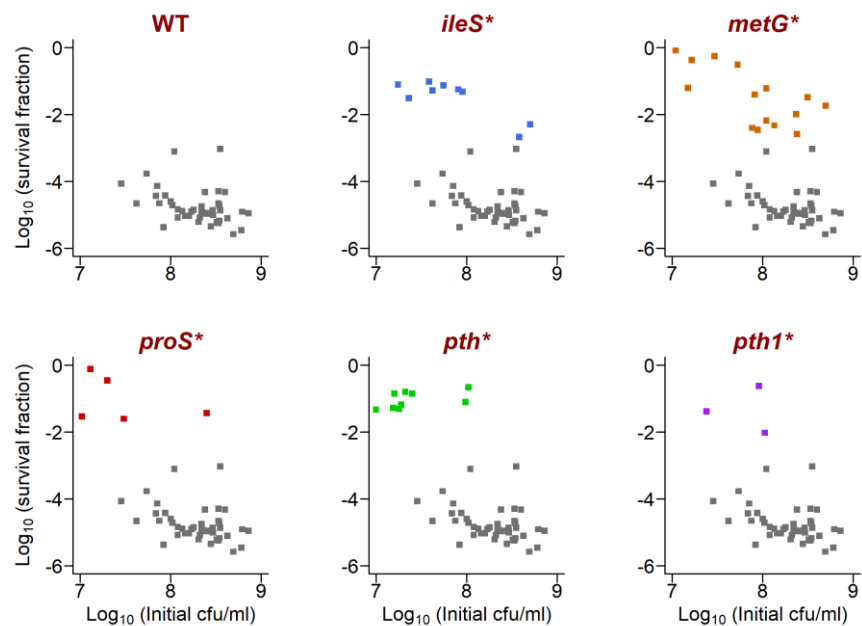

b.

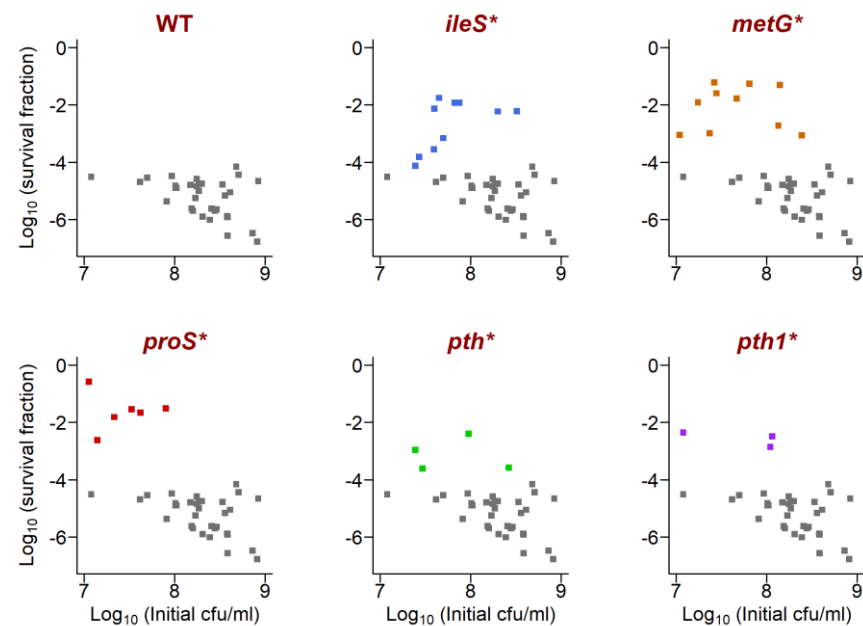

Supplement: FIG S2 [file mSystems.00847-19-sf002.pdf]

**Figure S3**

**a.**

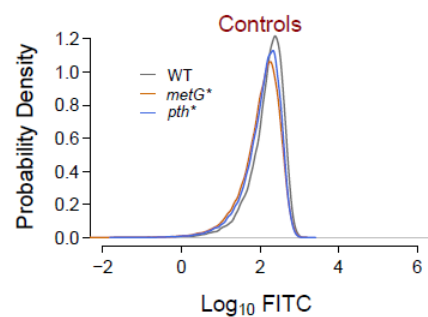

**b.**

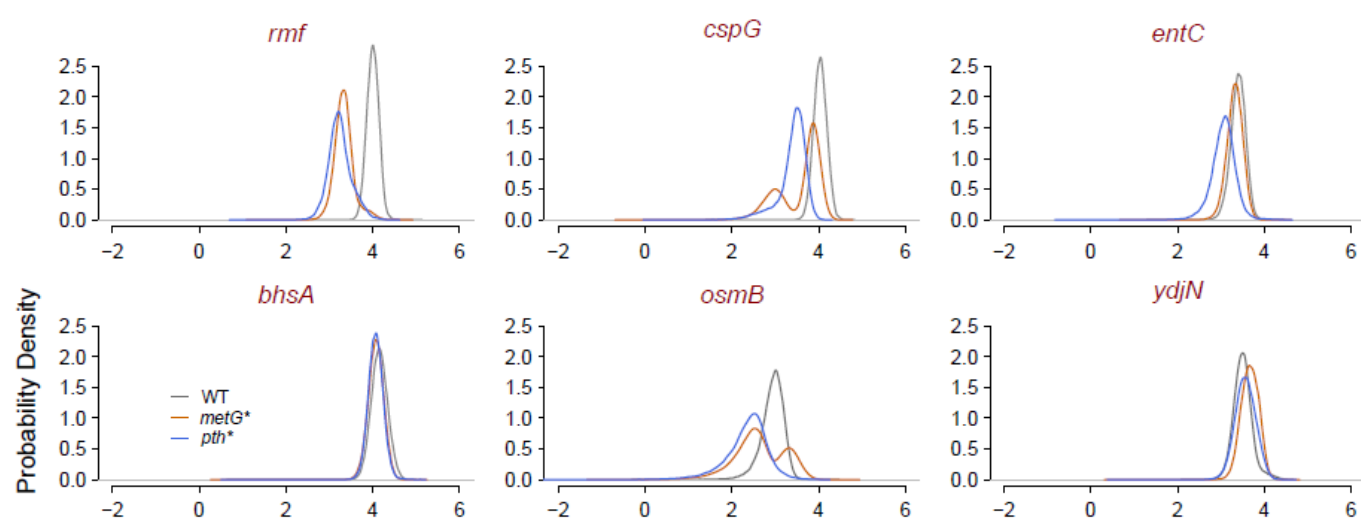

**c.**

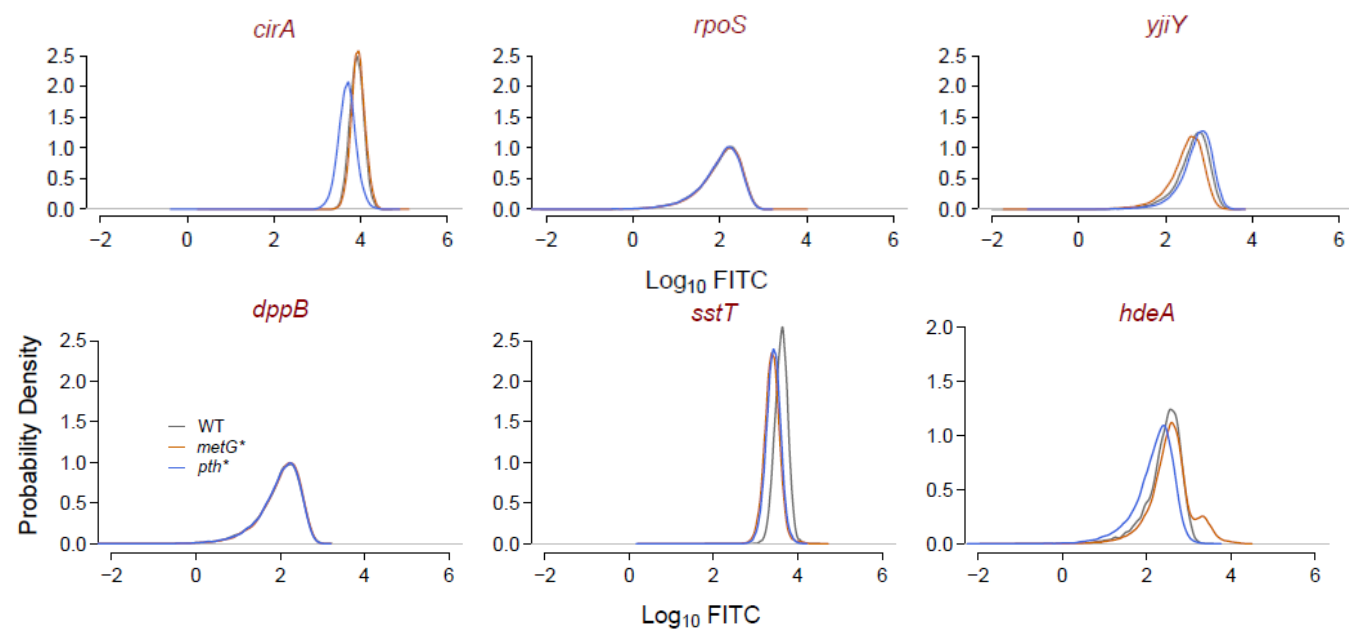

Supplement: FIG S3 [file mSystems.00847-19-sf003.pdf]

Figure S4

a.

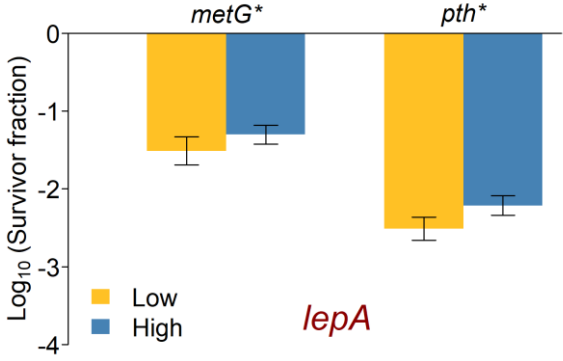

b.

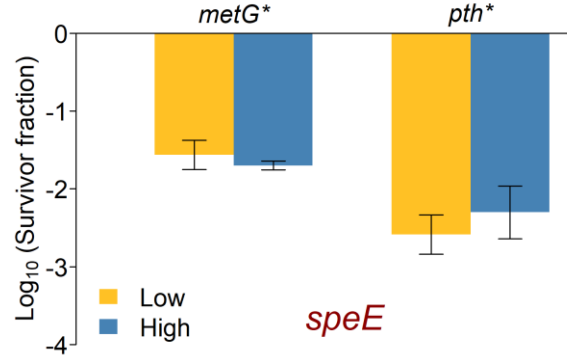

Supplement: FIG S4 [file mSystems.00847-19-sf004.pdf]
