# Supplementary material for: Extreme Antibiotic Persistence via Heterogeneity-Generating Mutations Targeting Translation
Source: mSystems. 2020 Jan 21;5(1):e00847-19. doi: 10.1128/mSystems.00847-19 (PMC6977076; doi:10.1128/mSystems.00847-19)
Supplement: TABLE S2 [file mSystems.00847-19-st002.pdf]

Table S2

Strain	Relative Ampicillin MIC (Mutant/WT)	Relative Ciprofloxacin MIC (Mutant/WT)	Doubling time (min)	Lag time (h)
WT	1	1	39.9 ± 1.45	1.13 ± 0.02
*leuS**	1	0.5 - 1	44.1 ± 2.51	8.34 ± 0.20
*pth**	0.5 - 1	1	43.1 ± 0.97	2.82 ± 0.04
*pth1**	1	0.5 - 1	39.2 ± 0.52	2.51 ± 0.06
*ileS**	1	0.5 - 1	45.7 ± 1.34	3.01 ± 0.19
*proS**	1 - 2	0.5	39.4 ± 0.18	1.71 ± 0.03
*metG**	0.5 - 1	0.5 - 1	39.6 ± 1.49	1.79 ± 0.06
