# Supplementary material for: Extreme Antibiotic Persistence via Heterogeneity-Generating Mutations Targeting Translation
Source: mSystems. 2020 Jan 21;5(1):e00847-19. doi: 10.1128/mSystems.00847-19 (PMC6977076; doi:10.1128/mSystems.00847-19)
Supplement: FIG S5 [file mSystems.00847-19-sf005.pdf]

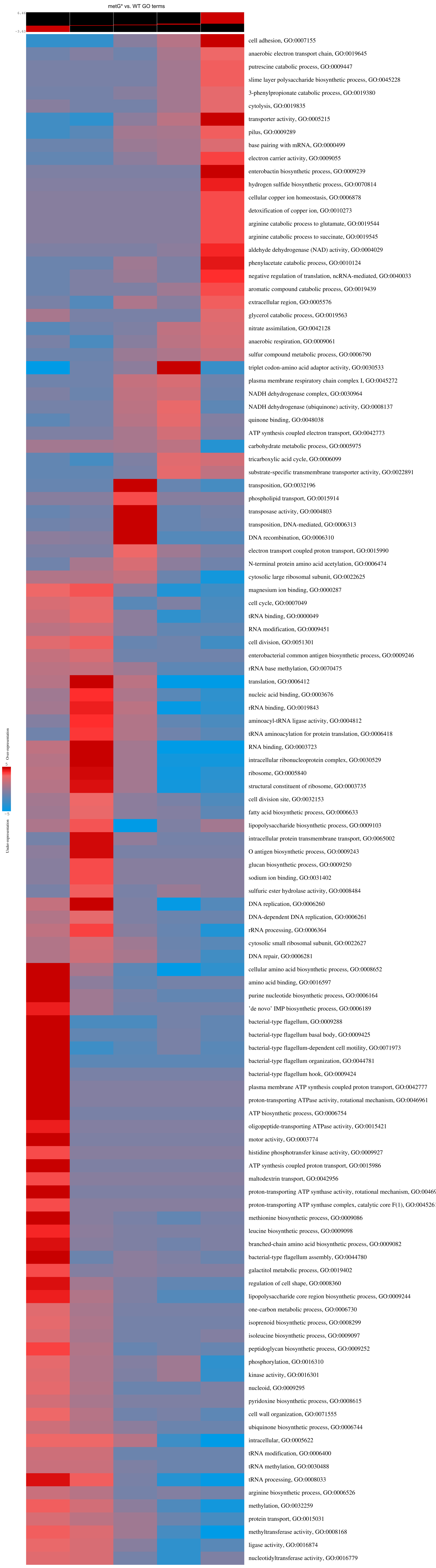
metG* vs. WT GO terms
6.46
-3.63
Over-representation
5
−5
Under-representation
cell adhesion, GO:0007155
anaerobic electron transport chain, GO:0019645
putrescine catabolic process, GO:0009447
slime layer polysaccharide biosynthetic process, GO:0045228
3-phenylpropionate catabolic process, GO:0019380
cytolysis, GO:0019835
transporter activity, GO:0005215
pilus, GO:0009289
base pairing with mRNA, GO:0000499
electron carrier activity, GO:0009055
enterobactin biosynthetic process, GO:0009239
hydrogen sulfide biosynthetic process, GO:0070814
cellular copper ion homeostasis, GO:0006878
detoxification of copper ion, GO:0010273
arginine catabolic process to glutamate, GO:0019544
arginine catabolic process to succinate, GO:0019545
aldehyde dehydrogenase (NAD) activity, GO:0004029
phenylacetate catabolic process, GO:0010124
negative regulation of translation, ncRNA-mediated, GO:0040033
aromatic compound catabolic process, GO:0019439
extracellular region, GO:0005576
glycerol catabolic process, GO:0019563
nitrate assimilation, GO:0042128
anaerobic respiration, GO:0009061
sulfur compound metabolic process, GO:0006790
triplet codon-amino acid adaptor activity, GO:0030533
plasma membrane respiratory chain complex I, GO:0045272
NADH dehydrogenase complex, GO:0030964
NADH dehydrogenase (ubiquinone) activity, GO:0008137
quinone binding, GO:0048038
ATP synthesis coupled electron transport, GO:0042773
carbohydrate metabolic process, GO:0005975
tricarboxylic acid cycle, GO:0006099
substrate-specific transmembrane transporter activity, GO:0022891
transposition, GO:0032196
phospholipid transport, GO:0015914
transposase activity, GO:0004803
transposition, DNA-mediated, GO:0006313
DNA recombination, GO:0006310
electron transport coupled proton transport, GO:0015990
N-terminal protein amino acid acetylation, GO:0006474
cytosolic large ribosomal subunit, GO:0022625
magnesium ion binding, GO:0000287
cell cycle, GO:0007049
tRNA binding, GO:0000049
RNA modification, GO:0009451
cell division, GO:0051301
enterobacterial common antigen biosynthetic process, GO:0009246
rRNA base methylation, GO:0070475
translation, GO:0006412
nucleic acid binding, GO:0003676
rRNA binding, GO:0019843
aminoacyl-tRNA ligase activity, GO:0004812
tRNA aminoacylation for protein translation, GO:0006418
RNA binding, GO:0003723
intracellular ribonucleoprotein complex, GO:0030529
ribosome, GO:0005840
structural constituent of ribosome, GO:0003735
cell division site, GO:0032153
fatty acid biosynthetic process, GO:0006633
lipopolysaccharide biosynthetic process, GO:0009103
intracellular protein transmembrane transport, GO:0065002
O antigen biosynthetic process, GO:0009243
glucan biosynthetic process, GO:0009250
sodium ion binding, GO:0031402
sulfuric ester hydrolase activity, GO:0008484
DNA replication, GO:0006260
DNA-dependent DNA replication, GO:0006261
rRNA processing, GO:0006364
cytosolic small ribosomal subunit, GO:0022627
DNA repair, GO:0006281
cellular amino acid biosynthetic process, GO:0008652
amino acid binding, GO:0016597
purine nucleotide biosynthetic process, GO:0006164
'de novo' IMP biosynthetic process, GO:0006189
bacterial-type flagellum, GO:0009288
bacterial-type flagellum basal body, GO:0009425
bacterial-type flagellum-dependent cell motility, GO:0071973
bacterial-type flagellum organization, GO:0044781
bacterial-type flagellum hook, GO:0009424
plasma membrane ATP synthesis coupled proton transport, GO:0042777
proton-transporting ATPase activity, rotational mechanism, GO:0046961
ATP biosynthetic process, GO:0006754
oligopeptide-transporting ATPase activity, GO:0015421
motor activity, GO:0003774
histidine phosphotransfer kinase activity, GO:0009927
ATP synthesis coupled proton transport, GO:0015986
maltodextrin transport, GO:0042956
proton-transporting ATP synthase activity, rotational mechanism, GO:00469
proton-transporting ATP synthase complex, catalytic core F(1), GO:004526
methionine biosynthetic process, GO:0009086
leucine biosynthetic process, GO:0009098
branched-chain amino acid biosynthetic process, GO:0009082
bacterial-type flagellum assembly, GO:0044780
galactitol metabolic process, GO:0019402
regulation of cell shape, GO:0008360
lipopolysaccharide core region biosynthetic process, GO:0009244
one-carbon metabolic process, GO:0006730
isoprenoid biosynthetic process, GO:0008299
isoleucine biosynthetic process, GO:0009097
peptidoglycan biosynthetic process, GO:0009252
phosphorylation, GO:0016310
kinase activity, GO:0016301
nucleoid, GO:0009295
pyridoxine biosynthetic process, GO:0008615
cell wall organization, GO:0071555
ubiquinone biosynthetic process, GO:0006744
intracellular, GO:0005622
tRNA modification, GO:0006400
tRNA methylation, GO:0030488
tRNA processing, GO:0008033
arginine biosynthetic process, GO:0006526
methylation, GO:0032259
protein transport, GO:0015031
methyltransferase activity, GO:0008168
ligase activity, GO:0016874
nucleotidyltransferase activity, GO:0016779

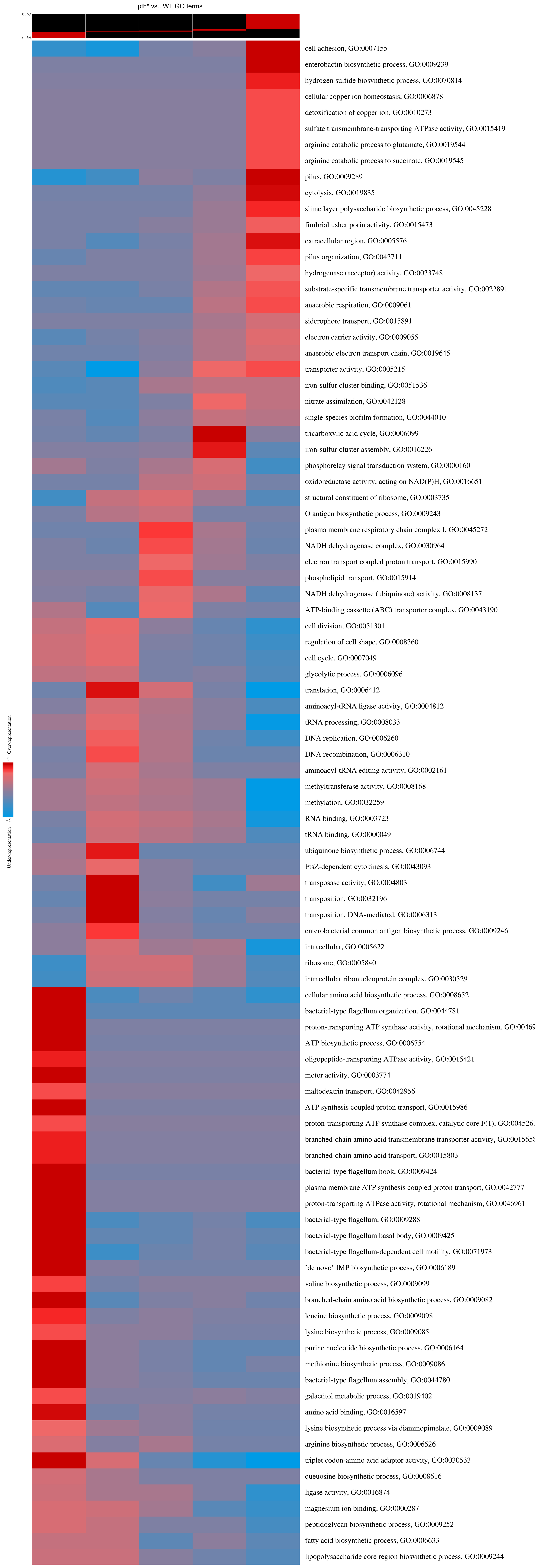
pth* vs.. WT GO terms
6.92
-2.44
Over-representation
5
-5
Under-representation
cell adhesion, GO:0007155
enterobactin biosynthetic process, GO:0009239
hydrogen sulfide biosynthetic process, GO:0070814
cellular copper ion homeostasis, GO:0006878
detoxification of copper ion, GO:0010273
sulfate transmembrane-transporting ATPase activity, GO:0015419
arginine catabolic process to glutamate, GO:0019544
arginine catabolic process to succinate, GO:0019545
pilus, GO:0009289
cytolysis, GO:0019835
slime layer polysaccharide biosynthetic process, GO:0045228
fimbrial usher porin activity, GO:0015473
extracellular region, GO:0005576
pilus organization, GO:0043711
hydrogenase (acceptor) activity, GO:0033748
substrate-specific transmembrane transporter activity, GO:0022891
anaerobic respiration, GO:0009061
siderophore transport, GO:0015891
electron carrier activity, GO:0009055
anaerobic electron transport chain, GO:0019645
transporter activity, GO:0005215
iron-sulfur cluster binding, GO:0051536
nitrate assimilation, GO:0042128
single-species biofilm formation, GO:0044010
tricarboxylic acid cycle, GO:0006099
iron-sulfur cluster assembly, GO:0016226
phosphorelay signal transduction system, GO:0000160
oxidoreductase activity, acting on NAD(P)H, GO:0016651
structural constituent of ribosome, GO:0003735
O antigen biosynthetic process, GO:0009243
plasma membrane respiratory chain complex I, GO:0045272
NADH dehydrogenase complex, GO:0030964
electron transport coupled proton transport, GO:0015990
phospholipid transport, GO:0015914
NADH dehydrogenase (ubiquinone) activity, GO:0008137
ATP-binding cassette (ABC) transporter complex, GO:0043190
cell division, GO:0051301
regulation of cell shape, GO:0008360
cell cycle, GO:0007049
glycolytic process, GO:0006096
translation, GO:0006412
aminoacyl-tRNA ligase activity, GO:0004812
tRNA processing, GO:0008033
DNA replication, GO:0006260
DNA recombination, GO:0006310
aminoacyl-tRNA editing activity, GO:0002161
methyltransferase activity, GO:0008168
methylation, GO:0032259
RNA binding, GO:0003723
tRNA binding, GO:0000049
ubiquinone biosynthetic process, GO:0006744
FtsZ-dependent cytokinesis, GO:0043093
transposase activity, GO:0004803
transposition, GO:0032196
transposition, DNA-mediated, GO:0006313
enterobacterial common antigen biosynthetic process, GO:0009246
intracellular, GO:0005622
ribosome, GO:0005840
intracellular ribonucleoprotein complex, GO:0030529
cellular amino acid biosynthetic process, GO:0008652
bacterial-type flagellum organization, GO:0044781
proton-transporting ATP synthase activity, rotational mechanism, GO:0046
ATP biosynthetic process, GO:0006754
oligopeptide-transporting ATPase activity, GO:0015421
motor activity, GO:0003774
maltodextrin transport, GO:0042956
ATP synthesis coupled proton transport, GO:0015986
proton-transporting ATP synthase complex, catalytic core F(1), GO:004526
branched-chain amino acid transmembrane transporter activity, GO:001565
branched-chain amino acid transport, GO:0015803
bacterial-type flagellum hook, GO:0009424
plasma membrane ATP synthesis coupled proton transport, GO:0042777
proton-transporting ATPase activity, rotational mechanism, GO:0046961
bacterial-type flagellum, GO:0009288
bacterial-type flagellum basal body, GO:0009425
bacterial-type flagellum-dependent cell motility, GO:0071973
'de novo' IMP biosynthetic process, GO:0006189
valine biosynthetic process, GO:0009099
branched-chain amino acid biosynthetic process, GO:0009082
leucine biosynthetic process, GO:0009098
lysine biosynthetic process, GO:0009085
purine nucleotide biosynthetic process, GO:0006164
methionine biosynthetic process, GO:0009086
bacterial-type flagellum assembly, GO:0044780
galactitol metabolic process, GO:0019402
amino acid binding, GO:0016597
lysine biosynthetic process via diaminopimelate, GO:0009089
arginine biosynthetic process, GO:0006526
triplet codon-amino acid adaptor activity, GO:0030533
queuosine biosynthetic process, GO:0008616
ligase activity, GO:0016874
magnesium ion binding, GO:0000287
peptidoglycan biosynthetic process, GO:0009252
fatty acid biosynthetic process, GO:0006633
lipopolysaccharide core region biosynthetic process, GO:0009244

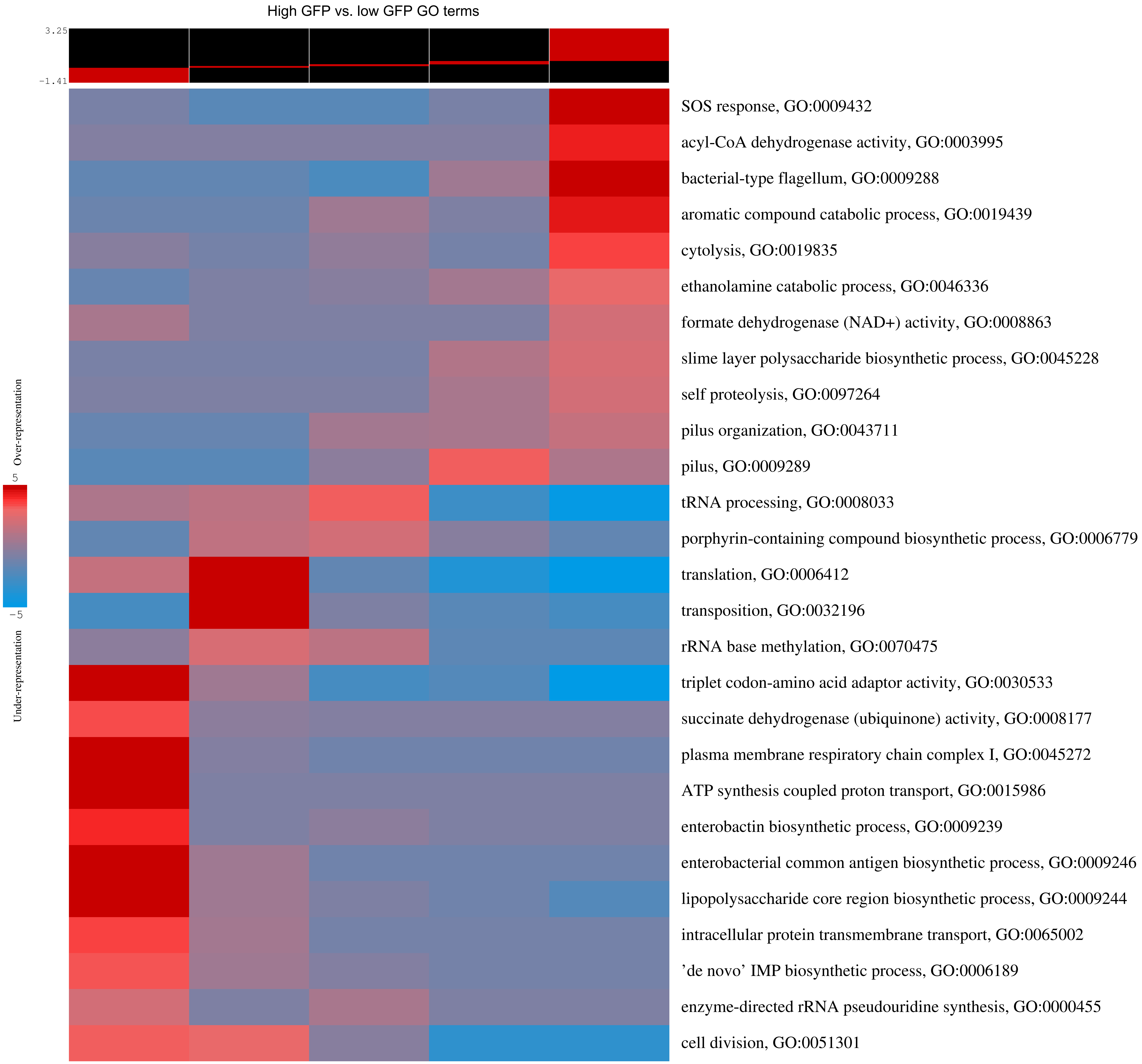

High GFP vs. low GFP GO terms
3.25
-1.41
Over-representation
5
−5
Under-representation
SOS response, GO:0009432
acyl-CoA dehydrogenase activity, GO:0003995
bacterial-type flagellum, GO:0009288
aromatic compound catabolic process, GO:0019439
cytolysis, GO:0019835
ethanolamine catabolic process, GO:0046336
formate dehydrogenase (NAD+) activity, GO:0008863
slime layer polysaccharide biosynthetic process, GO:0045228
self proteolysis, GO:0097264
pilus organization, GO:0043711
pilus, GO:0009289
tRNA processing, GO:0008033
porphyrin-containing compound biosynthetic process, GO:0006779
translation, GO:0006412
transposition, GO:0032196
rRNA base methylation, GO:0070475
triplet codon-amino acid adaptor activity, GO:0030533
succinate dehydrogenase (ubiquinone) activity, GO:0008177
plasma membrane respiratory chain complex I, GO:0045272
ATP synthesis coupled proton transport, GO:0015986
enterobactin biosynthetic process, GO:0009239
enterobacterial common antigen biosynthetic process, GO:0009246
lipopolysaccharide core region biosynthetic process, GO:0009244
intracellular protein transmembrane transport, GO:0065002
'de novo' IMP biosynthetic process, GO:0006189
enzyme-directed rRNA pseudouridine synthesis, GO:0000455
cell division, GO:0051301

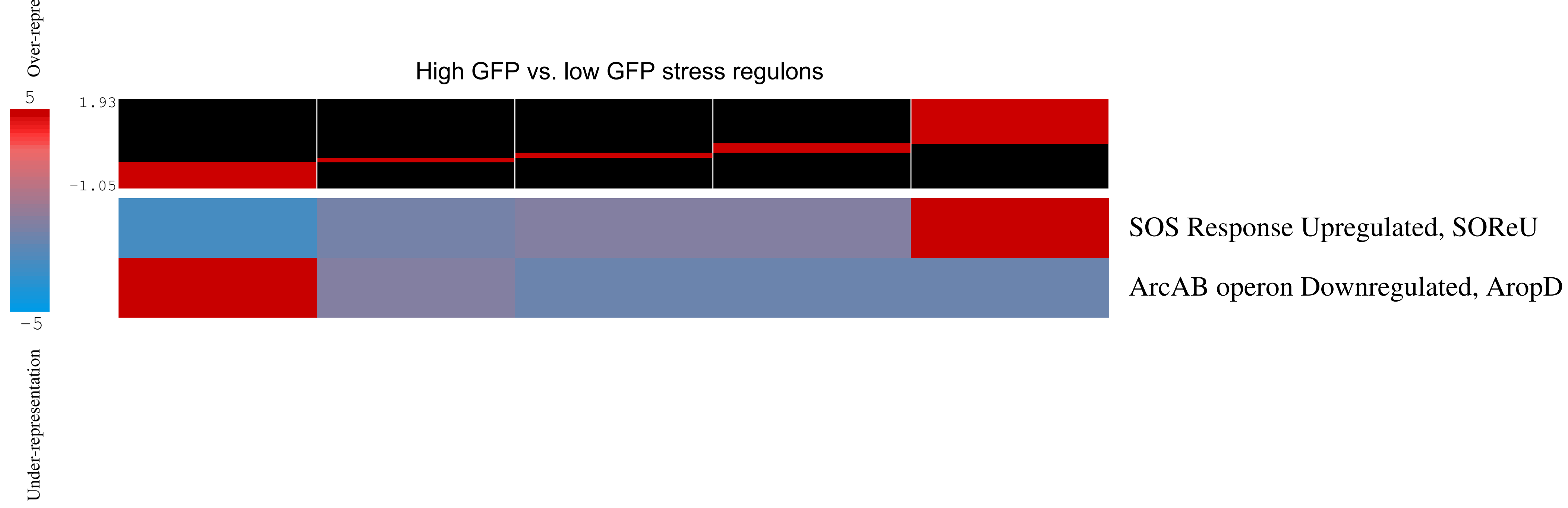
High GFP vs. low GFP stress regulons
Over-repre
5
-5
Under-representation
1.93
-1.05
SOS Response Upregulated, SOReU
ArcAB operon Downregulated, AropD
