# Supplementary material for: Extreme Antibiotic Persistence via Heterogeneity-Generating Mutations Targeting Translation
Source: mSystems. 2020 Jan 21;5(1):e00847-19. doi: 10.1128/mSystems.00847-19 (PMC6977076; doi:10.1128/mSystems.00847-19)
Supplement: TABLE S3 [file mSystems.00847-19-st003.pdf]

Table S3.

**Strain / Plasmid**	**Genotype**	**Source**
MG1655	WT *E. coli* K-12	ATCC 47076
Δ*10TA*	MG1655 Δ*mazF* Δ*chpB* Δ*relBE* Δ(*dinJ-yafQ*) Δ(*yefM-yoeB*) Δ*higBA* Δ(*prlF-yhaV*) Δ*yafNO* Δ*mqsRA* Δ*hicAB*	[1]
Δ*TA11*	Δ*10TA* Δ*hipBA*	This study
*leuS**	MG1655 *leuS**	This study
*selU**	MG1655 *selU**	This study
*metG**	MG1655 *metG**	This study
*pth**	MG1655 *pth**	This study
*pth1**	MG1655 *pth1**	This study
*ileS**	MG1655 *ileS**	This study
*proS**	MG1655 *proS**	This study
Δ*TA11 leuS**	Δ*TA11 leuS**	This study
Δ*TA11 selU**	Δ*TA11 selU**	This study
Δ*TA11 metG**	Δ*TA11 metG**	This study
Δ*TA11 pth**	Δ*TA11 pth**	This study
Δ*TA11 pth1**	Δ*TA11 pth1**	This study
Δ*TA11 ileS**	Δ*TA11 ileS**	This study
Δ*TA11 proS**	Δ*TA11 proS**	This study
pUA66-p-*rmf*		[2]
pUA66-p-*cspG*		[2]
pUA66-p-*entC*		[2]
pUA66-p-*bhsA*		[2]
pUA139-p-*osmB*		[2]
pUA66-p-*ydjN*		[2]
pUA139-p-*cirA*		[2]
pUA139-p-*rpoS*		[2]
pUA139-p-*yjiY*		[2]
pUA139-p-*dppB*		[2]
pUA139-p-*hdeA*		[2]
pUA66-p-*sstT*		[2]
pUA139-p-*lepA*		[2]
pUA66-p-*speE*		[2]
pCA24N		[3]
pCA24N-*rmf*		[3]
pCA24N-*osmB*		[3]
pCA24N-*cspG*		[3]

1. Maisonneuve E, Shakespeare LJ, Jørgensen MG, Gerdes K. Bacterial persistence by RNA endonucleases. Proceedings of the National Academy of Sciences. 2011;108(32):13206-11.
2. Zaslaver A, Bren A, Ronen M, Itzkovitz S, Kikoin I, Shavit S, et al. A comprehensive library of fluorescent transcriptional reporters for *Escherichia coli*. Nature Methods. 2006;3(8):623.
3. Kitagawa M, Ara T, Arifuzzaman M, Ioka-Nakamichi T, Inamoto E, Toyonaga H, et al. Complete set of ORF clones of *Escherichia coli* ASKA library (A Complete S et of E. coli K-12 ORF Archive): Unique Resources for Biological Research. DNA Research. 2005;12(5):291-9.

**Primer Description**	**Primer sequence**[a]
Forward primer for deletion of the *hipBA* locus	ACTTATA ATATCC CCTTAAGCGG ATAAACTTGC TGTGGACGTA TGACATG gtgtaggctggagctgcttc[b]
Reverse primer for deletion of the *hipBA* locus	GGCGGTCATG ATT GTC ATG CTC ATT AAC AAT GAC CAA ACC CCA TAT CTC A catatgaatatcctccttag[b]
Forward primer for *leuS**	**TCGGTACCCGGGGATCGC** AGC TCA TGG CGA ACT CTT C
Reverse primer for *leuS**	**CCGGTCGACTCTAGAG** CCG CCA GAG ATA ACA ACA ATG
Forward primer for *selU**	**TCGGTACCCGGGGATCGC** GCG GCA AAG CGG TAG AAG
Reverse primer for *selU**	**CCGGTCGACTCTAGAG** GGC CAT GTG TCA GAT CAA G
Forward primer for *metG**	**TCGGTACCCGGGGATCGC** ACC TGG CCT CCC GTA ATG
Reverse primer for *metG**	**CCGGTCGACTCTAGAG** GGT GGA TAA GTC CGC TAC
Forward primer for *pth**	**TCGGTACCCGGGGATCGC** TTA TGT GCG GGC GTG ATC
Reverse primer for *pth**	**CCGGTCGACTCTAGAG** TGG AAA GTT GGC CGC TTC
Forward primer for *pth1**	**TCGGTACCCGGGGATCGC** GGG CCG CAA CCA GTA AAC
Reverse primer for *pth1**	**CCGGTCGACTCTAGAG** AAA GCA GCG AAC AAC GTG
Forward primer for *ileS**	**TCGGTACCCGGGGATCGC** TTG CGC GTG ATG AAT TAA C
Reverse primer for *ileS**	**CCGGTCGACTCTAGAG** ACT CAT CAG GCA AAC TTA C
Forward primer for *proS**	**TCGGTACCCGGGGATCGC** TCT CCC CTT TGC CGA ATC
Reverse primer for *proS**	**CCGGTCGACTCTAGAG** TAC GCC TTT GGT TTA TCC

a The sequence in **bold** overlaps with the pKOV plasmid, and is required for the Gibson Assembly.
b The bases in the UPPERCASE are homologous to regions flanking the *hipBA* locus, and the bases in the lowercase target the Kanamycin cassette in the pKD4 plasmid.
